# Multidimensional evaluation of voice outcomes following total laryngectomy: a prospective multicenter cohort study

**DOI:** 10.1007/s00405-020-06216-z

**Published:** 2020-07-21

**Authors:** Klaske E. van Sluis, Rob J. J. H. van Son, Lisette van der Molen, Anthony John MCGuinness, Carsten E. Palme, Daniel Novakovic, Danielle Stone, Lydia Natsis, Emma Charters, Kelly Jones, Richard Dirven, Michiel W. M. van den Brekel

**Affiliations:** 1 Department of Head and Neck Oncology and Surgery, Netherlands Cancer Institute-Antoni van Leeuwenhoek, Plesmanlaan 121, 1066CX Amsterdam, The Netherlands; 2 Amsterdam Center for Language and Communication, University of Amsterdam, Amsterdam, The Netherlands; 3 Head and Neck Department, Department of Surgery, Chris O’Brien Lifehouse Hospital, Sydney, NSW Australia; 4 Department of Head and Neck Surgery, Royal Prince Alfred Hospital, Sydney, NSW Australia; 5 Department of Surgery, Ear Nose & Throat, Westmead Hospital, Westmead, NSW Australia; 6 Department of Ear, Nose and Throat, Head and Neck Surgery, Liverpool Hospital, Liverpool, NSW Australia; 7 Department of Otolaryngology, Head and Neck Surgery, Faculty of Medicine and Health, University of Sydney, Sydney, NSW Australia

**Keywords:** Total laryngectomy, Voice outcome, Acoustics, Perceptual, Patient-reported

## Abstract

**Purpose:**

The purpose of this study is to assess the general course of acoustic, patient rated, and clinician-rated voice outcomes from pre- up to 12 months post total laryngectomy.

**Methods:**

Patients admitted to a total laryngectomy in five participating hospitals in Australia and The Netherlands were included. Assessments took place at pre-, 3 months, 6 months, and 12 months post-surgery. Voice outcomes are evaluated with the Acoustic Voice Quality Index (AVQI), perceptual scales, and patient-reported outcome measures including VHI-10 and EQ-5D-5L. Statistical analyses include descriptive statistics, *t* tests (pre- to 6 months post-surgery), Linear Mixed Effect models.

**Results:**

The study included 43 participants. A significant worsening of *AVQI* is seen from pre- to post-surgery evaluated with *t* test (p < 0.001). The Linear Mixed Effect model confirmed *Time* as a significant factor in predicting *AVQI* score (*p* ≤ 0.001), as well as *perceptual rated voice quality* by the clinician (*p* = 0.015) and *patient*-*reported perceptual rated voice quality* (*p* = 0.002). No statistical significance was found in *VHI*-*10* scores over time.

**Conclusion:**

Successful TE-speech was achieved in most participants, some had to rely on augmentative alternative communication methods. Patient-reported outcomes indicate acceptance of the condition and sufficient coping in the long term. However, acoustic rated voice quality is abnormal at all post-surgery time-points. *AVQI* proved to be a useful instrument to evaluate TE-speech. There is a need for validation and determination of cut-off values for *VHI*-*10* and *AVQI* for use in TE-speech.

## Introduction

One of the most important rehabilitation goals after total laryngectomy is voice rehabilitation. To compensate for the loss of voice, patients ideally rehabilitate speech with a voice prosthesis, so called Tracheo-Esophageal Speech (TE-speech) [1–3]. If this is not possible, alternative communication methods include esophageal speech, electrolarynx speech, or augmentative alternative communication. Successful TE-speech after laryngectomy is not guaranteed as outcomes in intelligibility, voice quality, and experienced voice handicap varying vary strongly between laryngectomized patients.

To evaluate voice outcomes, it is recommended to use multi-dimensional analysis which combines objective and subjective outcome measures [4]. Voice recordings of connected speech and sustained phonation can be used to objectively measure voice outcomes with acoustic analysis, focusing on pitch, perturbation, and harmonics-to-noise ratio. The Acoustic Voice Quality Index (AVQI) is a widely used measure reflecting a number of acoustic outcomes in one score [5, 6]. Subjective measures, on the other hand, include clinician and patient-rated perceptual evaluation of voice and speech, and Patient-Reported Outcome Measures (PROMs) assessing Quality of Life (QoL) and speech-related QoL.

Little is known about the course of voice outcomes in the first year after surgery [2]. Present studies, prospectively assessing the course of QoL and reported voice problems, demonstrated that, in the long term, health related QoL and speech-related QoL improve post-surgery compared to pre-laryngectomy [7–12]. Before laryngectomy QoL is often lower compared to the reference standard due to initial organ preservation treatment or by the tumour itself [7, 8]. Immediately after laryngectomy QoL scores drop even further. The following year after surgery some patients recover back to baseline whilst some do not recover [7, 8]. For longitudinal QoL studies there is a significant selection bias, as patients whose health problems prevent their participation through the duration of the study are often excluded from study analysis, which may result in over optimistic QoL outcomes [7, 13–15]. Studies reporting acoustic voice outcome after total laryngectomy often compare different groups of voice restoration methods, and most report only on sufficient or even excellent speakers, potentially leading to selection bias [2, 16–18]. Despite this, several studies have demonstrated that poorer speech-related QoL is associated with lower health-related QoL scores after total laryngectomy [19, 20].

Prospective multidimensional evaluation overall groups of substitute-voice-speakers after laryngectomy has not yet been described in the literature. This study aims to assess the change of acoustic, patient rated, and clinician-rated voice outcomes from pre-up to 12 months post-surgery. These outcomes could potentially play a role for both patients and clinicians to assist them in counselling and decision-making regarding treatment and rehabilitation.

## Methods

### Study design

A prospective multicenter design was conducted over five hospitals. Data was collected between April 2015 till May 2019 in the following institutes: [xxx], [xxx], [xxx], [xxx], [xxx]. Ethical clearance was obtained for the [xxx] (number [xxx]) as well for [xxx] (Protocol [xxx]).

Patients eligible for total laryngectomy were approached to participate. Inclusion criteria were: over 18 years of age, curative intent laryngectomy, physically and cognitively able and willing to perform assessments. Informed consent was obtained from all participants. When participants during the course of the study were palliated or died, follow-up assessments were cancelled.

Data were collected at four time-points for each participant: prior to total laryngectomy (T0), 3 months (T1), 6 months (T2), and 12 months (T3) post-surgery. Study assessments included perceptual evaluation, voice recordings and patient-reported outcome measures (PROMs). Data collection was performed by an experienced Speech Language Pathologist (SLP). Voice recordings included reading aloud a text, phonation of the vowel/a/at normal pitch, as well as low, high, soft and loud.

Visual Analogue Scales (VAS) were used to perceptual rate voice quality, resulting in a score of 0 to 10, with 0 representing worst and 10 indicating the best voice quality. Perceptual scores were provided by the clinician as well as the participant, resulting in the variables *Perc. Voice SLP* and *Perc. Voice Pt*. The use of this VAS perceptual scales are derived from a dedicated perceptual rating scale for substitute voices [21].

PROMs consisted *EQ*-*5D*-*5L* and *Voice Handicap Index 10 item version* (*VHI*-*10*). The *EQ*-*5D*-*5L* is a validated patient-report questionnaire that assesses a patient’s current health-related QoL [22]. It consists of 5 dimensions: mobility, self-care, daily activities, pain/discomfort and anxiety/depression. The final continuous outcome ranges from 0 to 1, a higher score indicates better health-related QoL [22]. Scores were interpreted with the Dutch country-specific reference values [23]. The *VHI*-*10* assesses experienced voice handicap [24]. *VHI*-*10* includes ten questions covering three sub-themes: functional, physical and emotional. The total *VHI*-*10* continuous outcome is a score ranging from 0 to 40, a higher score indicates a greater handicap. Scores above 11 are considered as abnormal [20, 24–26]. The *VHI*-*10* is a widely used and validated questionnaire, although not specifically validated for use after total laryngectomy.

### Demographics and oncological history

Demographics and oncological history were collected during the first and second assessment. Demographic variables included sex and age at the time of surgery. Oncological history included tumour site, T-stage, and N-stage as defined by the pathologist post-surgery, timing of (chemo)radiotherapy, and primary, functional or salvage indication for total laryngectomy. Surgery specific data included neck dissection, neopharyngeal reconstruction, tongue base resection, myotomy of the upper esophageal sphincter, primary voice prosthesis placement, and secondary voice prosthesis placement. In the finalizing phase of the study the variables were checked with information retrieved from the local data desk.

### Acoustic analysis

Segmentation, acoustic analysis, and obtaining AVQI scores is performed using Praat [27]. The main outcome measure Acoustic Voice Quality Index (AVQI) requires recordings of a sustained vowel/a/and a read aloud text [5, 6]. Sustained/a/sounds of at least 3 s were used. If no single realization of 3 s was available, realizations were concatenated. From the read aloud text or read aloud sentences 4 s of connected speech was used. If these included long pauses, these were removed. The AVQI algorithm includes the cepstral peak prominence, harmonics-to-noise ratio, shimmer local, shimmer local dB, as well as the slope and tilt of the regression line through the long-term average spectrum. When incorporated into Praat, the analysis script estimates an AVQI score, which ranges from 0 to 10. A lower score indicates a better voice quality, > 2.95 is the cut-off point, scores above are indicated as distorted. Participants who were unable to produce voice post-surgery but did perform the assessment were rated with an AVQI score of 10.

### Statistical analysis

The data is analysed with the help of IBM SPSS software to perform descriptive statistics [28] and R [29] for inferential statistics and modelling. No sample size calculation was performed since numbers of inclusion were based on the available patients admitted to TL.

Study sample characteristics were tabulated and visualized. Primary outcomes were *VHI*-*10*, *AVQI*, *Perc. Voice SLP*, and *Perc. Voice Pt.* Paired *t* tests between T0 and T2 were performed with a statistical significance level set at *p* ≤ 0.05. To investigate treatment variables, three oncologic treatment variables were transformed to dichotomous variables, including (a) primary surgical treatment vs. salvage surgical treatment, (b) primary closure vs. major reconstruction of the neopharynx, and (c) a History of CRT vs. RT. The variable (c) History of CRT vs. RT proved to be redundant and was dropped. Definitions of the definite chosen variables are shown in Table 1.

**Table 1:** Transformed oncological treatment factors into dichotomous variables

Variable	Includes participants with
Treatment	
Primary surgical treatment	Total laryngectomy as a primary cancer treatment
	Total laryngectomy as a treatment for a secondary primary tumour
Salvage surgical treatment	Total laryngectomy as a salvage treatment in case of recurrent disease
	Total laryngectomy as a treatment for a dysfunctional larynx
Reconstruction	
Primary closure	Primary closure of the neopharynx
Major reconstruction	Major reconstruction of the neopharynx with the use of flaps including a pectoralis major-flap, free flap or gastric pull up

Correlations between primary outcome measures are investigated using linear mixed effect models with (pseudo) *R*^2^ and Chi square ANOVA on *Y* ~ *X* +(1|Subject) + (1|*T*) against *Y* ~ 1 +(1|Subject) + (1|*T*). Scatter plots are made for visualization (Appendix I, Appendix II). Because of multiple testing we used Bonferroni correction and adapted alpha to ≤ 0.01.

To estimate the importance of the factors studied for outcomes in *VHI*-*10, AVQI*, and *perceptual* rated voice quality over time, linear mixed effect models were created (Appendix III, Appendix IV, Appendix V). The model analyses the relationship between *AVQI*, *VHI*-*10*, and *Perc. Voice SLP* on the one hand and the fixed effects *Time* (T0, T1, T2, T3), *Treatment* (primary surgery vs. salvage), and *Reconstruction* (Primary closure vs. Major reconstruction) on the other hand.

## Results

### Study sample

**Fig. 1:**
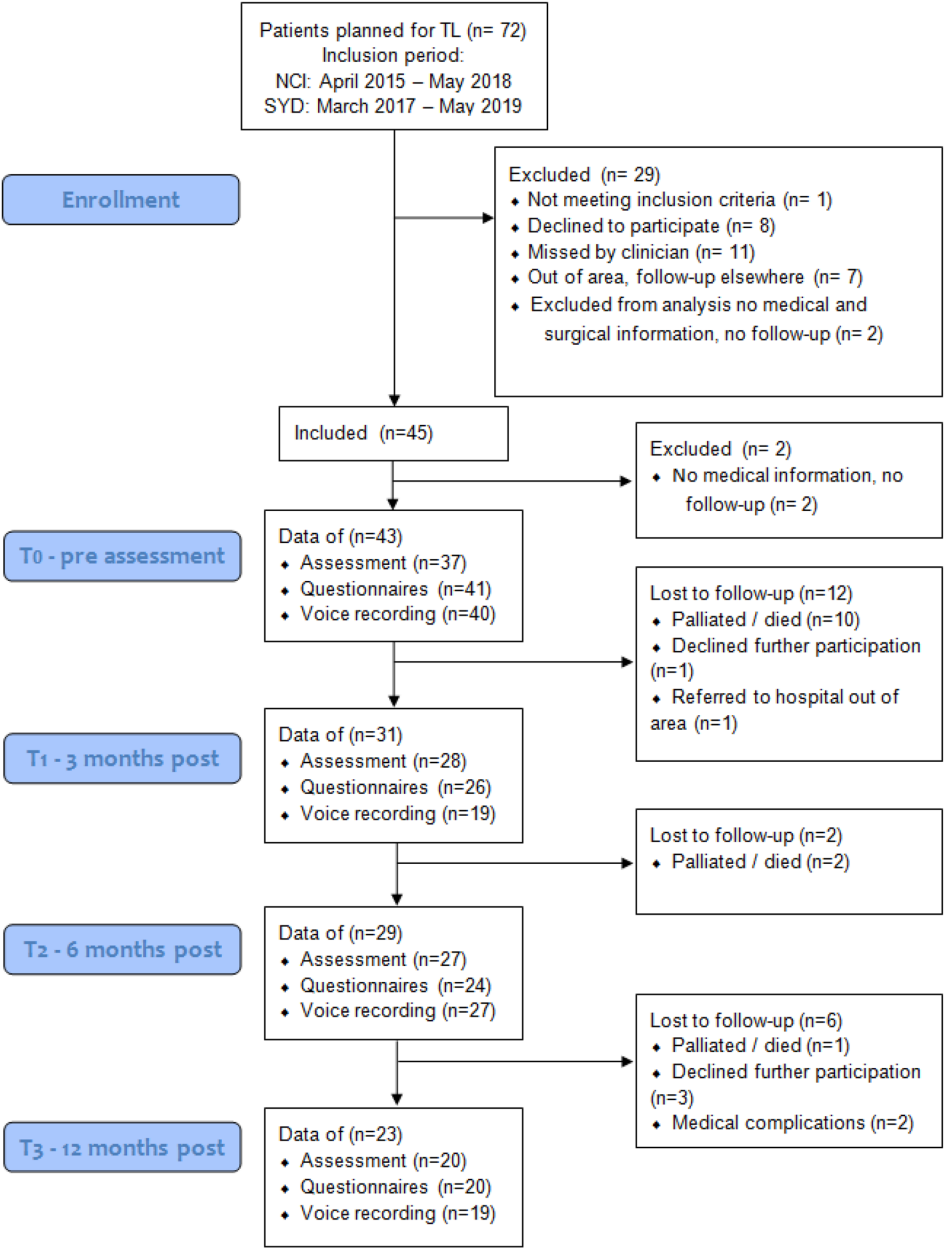
Flow-chart of study inclusion and follow-up of participants

Inclusion, follow-up, and availability of data at the assessments are shown in the flow chart in Fig. 1. Overall sites there was a total of 72 possible candidates who underwent total laryngectomy in the study time frame, of whom 43 were included in the study. Thirty-four from the Netherlands, nine from the Australian sites. Reasons for exclusion were: decline to participate (*n *= 8), live out of the area (*n *= 7), missed by the clinician (*n *= 11), total laryngectomy in combination with total glossectomy (*n *= 1), no medical information and follow-up assessments available (*n *= 2).

Patient characteristics are shown in Table 2. The majority of the included participants were male (*n *= 33; 77%), mean age was 64 years old at the time of surgery (range 43–84). For 19 participants (44%) the total laryngectomy was the primary surgical treatment, in 24 cases 56% total laryngectomy was a salvage treatment. In 16 participants (37%) of the cases primary closure of the neopharynx was performed, major reconstruction was needed in 27 (63%) of the cases. The Australian patients (*n *= 9) did not differ substantially from the Dutch. Within the Australian group, all patients were male, for 33% (*n *= 3) total laryngectomy was the primary treatment, 78% (*n *= 7) had a major reconstruction of the neopharynx.

**Table 2:** Demographic and clinical characteristics of the study population *n *= 43

Variables	*n*	%
Sex		
Male	33	76.7
Female	10	23.3
Tumour site		
Larynx	31	72.1
Hypopharynx	11	25.6
Other	1	2.3
Initial T-stage		
T1	4	9.3
T2	7	16.3
T3	13	30.2
T4	19	44.2
Initial N-stage		
N0	23	53.5
N+	20	46.5
Tracheostomy before TL		
Yes	16	37.2
No	27	62.8
(Chemo)radiotherapy		
Chemo-radiotherapy	10	23.3
Radiotherapy before TL	21	48.8
Radiotherapy after TL	11	25.6
No (chemo)radiotherapy	1	2.3
Indication for TL		
Primary surgical treatment	19	44.2
Primary cancer	11	25.6
Secondary primary cancer	8	18.6
Salvage surgical treatment	24	55.8
Recurrence of disease	16	37.2
Dysfunctional larynx	8	18.6
Neopharynx reconstruction		
Primary closure	16	37.2
Major reconstruction	27	62.8
Pectoralis major flap	14	32.6
Free flap	10	23.3
Gastric pull-up	3	7
Neck dissection		
No	14	32.6
Yes	29	67.4
Tongue base resection		
No	36	83.7
Yes	7	16.3
Myotomy		
No	16	37.2
Yes	27	62.8
Primary voice prosthesis placement		
No	9	20.9
Yes	34	79.1
Secondary voice prosthesis placement		
No	3	7
Yes	6	14
Not applicable	34	79.1
Died within year after surgery		
No	28	72.1
Yes	13	23.3
Unknown	2	4.6

*TL*Total laryngectomy

Before surgery 16 participants (37%) had a tracheostomy, which influences their communicative abilities. Although a high number of participants received a voice prosthesis, satisfactory voice rehabilitation with TE-speech was not accomplished in all cases. Methods of communication are tabulated in Table 3. In total, 93% of the participants received a voice prosthesis, 79% (*n *= 34) received primary puncture, 14% (*n *= 6) secondary puncture. Seven percent (*n *= 3) did not receive a voice prosthesis. Verbal communication with TE-speech was documented in 17 out of 27 participants at T1, 22 out of 25 participants at T2, and 20 out of 22 participants T3.

**Table 3:** Communication method used post total laryngectomy during follow-up for the total group of participants (*n *= 43)

	3 m post	6 m post	12 m post
	*n* (%)	*n* (%)	n (%)
Tracheo-esophageal speech	17 (39.5)	22 (51.1)	20 (46.5)
Electrolarynx speech	2 (4.7)	1 (2.3)	0 (0)
Esophageal speech	0 (0)	0 (0)	0 (0)
Augmentative alternative communication	8 (18.6)	2 (4.7)	2 (4.7)
Lost to follow-up	12 (27.9)	14 (32.5)	20 (46.5)
Missing	4 (9.2)	4 (9.2)	1 (2.3)

Of the total group, 30% (*n *= 13) died within the first year after surgery and were excluded from the analysis. Two-thirds of this group (*n *= 9) did not achieve TE-speech, they had to depend on augmentative alternative communication. Most of this group (*n *= 7) did receive a voice prosthesis but could not use this due to postoperative complications such fistulas, only two participants in this group did not receive a voice prosthesis due to medical issues.

### General course of self-reported outcomes and acoustic voice quality

**Fig. 2:**
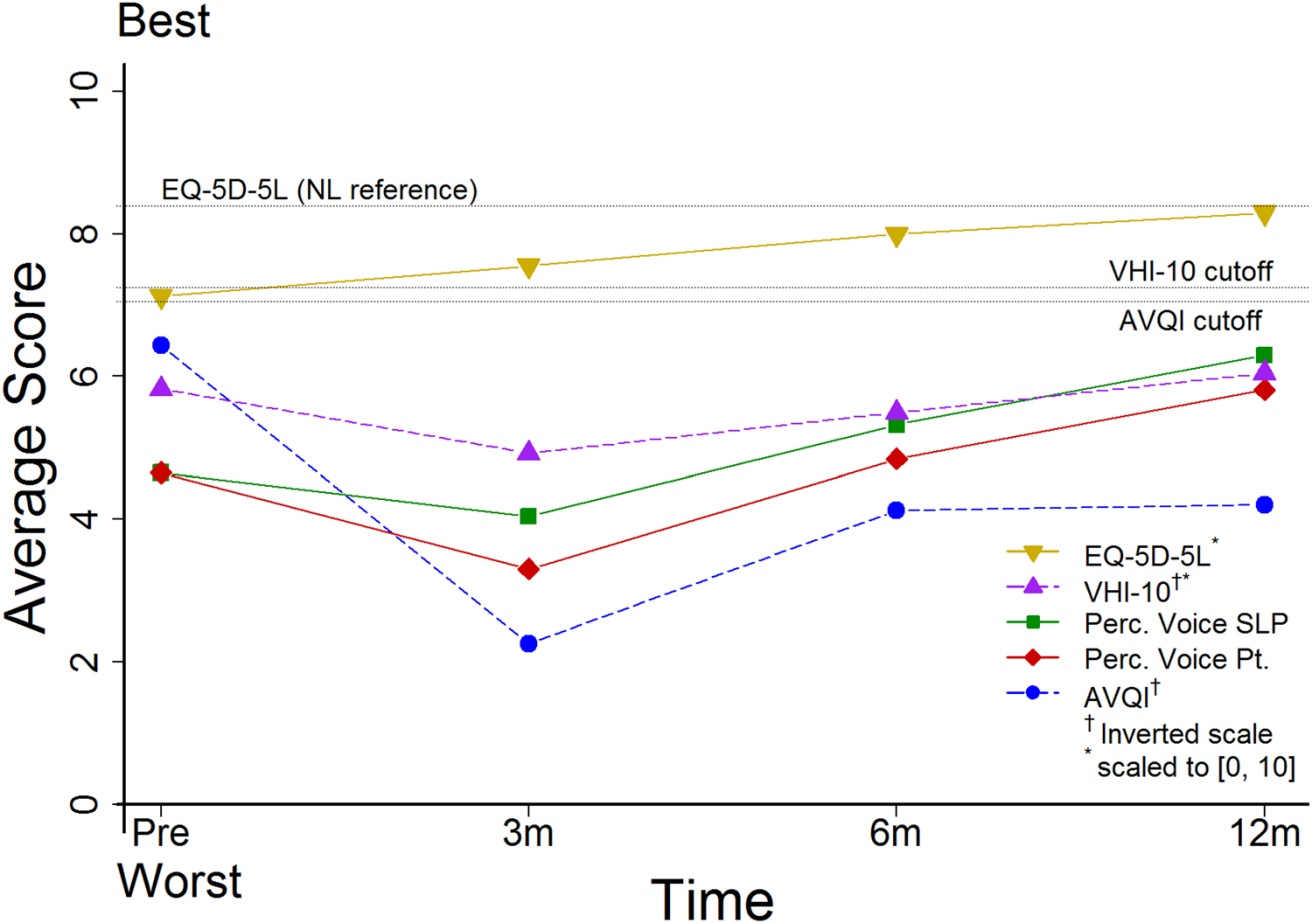
Graph visualizing mean scores for the total group for *EQ*-*5D*-*5L*, *VHI*-*10*, *AVQI* scores, Perc. Voice SLP, and Perc. Voice Pt. at each time point. *EQ*-*5D*-*5L* and *VHI*-*10* scaled 0–10, *AVQI* and *VHI*-*10* inverted. For easier interpretation, we inverted and scaled all outcome measures 0–10. Abbreviations: *EQ*-*5D*-*5L*: scores are obtained with the EQ-5D-5L and range from 0 to 1, *AVQI:* Acoustic Voice Quality Index (range 0–10); Perc. Voice SLP: Perceptual rated voice quality by the SLP (visual analogue scale 0–10); Perc. Voice Pt: Perceptual rated voice quality by the participant (visual analogue scale 0–10)

Mean scores for *EQ*-*5D*-*5L*, *VHI*-*10*, *AVQI*, *Perc. Voice SLP*, and *Perc. Voice Pt* for the total group and the defined sub-groups for the time points T0 (pre-surgery), T1 (3 months post), T2 (6 months post), and T3 (12 months post) are shown in Table 4. Primary outcome measures show high variation, which is demonstrated by the large standard deviation provided in Table 4. Figure 2 shows the course over time, scaled 0–10, a higher score indicating better outcome. After surgery, a worsening of all voice related values is seen, which gradually improves over time. Statistical significance was reached for *AVQI* (*p* < 0.001) for the difference of T0 to T2, for the other outcome measures no statistically significant difference was found with paired *t* test.

**Table 4:** Primary outcome measures at T0, T1, T2, and T3 for the total group of participants and sub groups including indication for total laryngectomy and type of neopharynx reconstruction

	T0—pre-surgery		T1—3 months post		T2—6 months post		T3—12 months post	
	*n*	Mean (SD)	*n*	Mean (SD)	*n*	Mean (SD)	*n*	Mean (SD)
*EQ*-*5D*-*5L*								
Total group	40	0.712 (0.203)	26	0.755 (0.151)	22	0.799 (0.169)	23	0.830 (0.164)
Primary surgical treatment	18	0.768 (0.168)	12	0.807 (0.171)	10	0.859 (0.181)	12	0.888 (0.114)
Salvage surgical treatment	22	0.667 (0.222)	14	0.711 (0.122)	12	0.749 (0.147)	11	0.766 (0.190)
Primary closure neopharynx	15	0.717 (0.266)	12	0.773 (0.152)	10	0.772 (0.224)	9	0.831 (0.117)
Major reconstruction neopharynx	25	0.710 (0.160)	14	0.739 (0.156)	12	0.823 (0.111)	14	0.829 (0.193)
*VHI*-*10*								
Total group	38	16.7 (10.6)	25	20.3 (10.0)	23	18.0 (8.9)	22	15.8 (12.0)
Primary surgical treatment	17	18.7 (8.6)	11	15.9 (10.2)	12	15.1 (9.1)	11	15.0 (11.1)
Salvage surgical treatment	21	15.1 (12.0)	14	23.8 (8.6)	11	21.3 (7.8)	11	16.6 (13.3)
Primary closure neopharynx	15	17.2 (11.1)	12	18.0 (11.0)	10	13.2 (6.9)	9	11.9 (8.8)
Major reconstruction neopharynx	23	16.4 (10.6)	13	22.5 (8.8)	13	21.8 (8.6)	13	18.5 (13.4)
*AVQI*								
Total group	37	3.57 (1.69)	21	8.07 (2.77)	24	5.97 (1.73)	16	5.99 (2.94)
Primary surgical treatment	17	3.48 (1.79)	8	8.41 (3.10)	13	6.46 (2.16)	9	5.45 (2.85)
Salvage surgical treatment	20	3.65 (1.63)	13	7.86 (2.66)	11	5.38 (0.75)	7	6.67 (3.12)
Primary closure neopharynx	13	3.56 (1.83)	9	7.15 (2.97)	13	5.24 (0.77)	6	4.77 (1.22)
Major reconstruction neopharynx	24	3.58 (1.64)	12	8.76 (2.51)	11	6.82 (2.16)	10	6.72 (3.46)
*Perc. Voice SLP*								
Total group	37	4.65 (2.58)	27	4.04 (3.26)	25	5.32 (2.87)	22	6.30 (2.69)
Primary surgical treatment	15	3.80 (2.62)	12	4.25 (2.96)	12	5.38 (2.48)	12	6.54 (2.23)
Salvage surgical treatment	22	5.23 (2.44)	15	3.87 (3.58)	13	5.27 (3.30)	10	6.00 (3.27)
Primary closure neopharynx	13	4.85 (2.91)	12	4.50 (3.56)	11	5.86 (2.17)	8	7.13 (1.36)
Major reconstruction neopharynx	24	4.54 (2.45)	15	3.67 (3.09)	14	4.89 (3.34)	14	5.82 (3.17)
*Perc. Voice Pt*								
Total group	37	4.65 (2.63)	27	3.30 (3.01)	25	4.84 (2.39)	21	5.81 (2.80)
Primary surgical treatment	15	3.93 (2.49)	12	3.42 (3.61)	12	5.08 (2.47)	12	6.08 (2.97)
Salvage surgical treatment	22	5.14 (2.66)	15	3.20 (2.57)	13	4.62 (2.40)	9	5.44 (2.70)
Primary closure neopharynx	13	2.23 (2.35)	12	4.08 (3.45)	11	5.73 (2.15)	7	6.14 (2.55)
Major reconstruction neopharynx	24	4.33 (2.76)	15	2.67 (2.55)	14	4.14 (2.41)	14	5.64 (3.00)

*T0*, pre-laryngectomy; *T1*, 3 months post-laryngectomy; *T2*, 6 months post-laryngectomy; *T3*, 12 months post-laryngectomy. *EQ*-*5D*-*5L*: scores are obtained with the EQ-5D-5L and range from 0 to 1, with a higher score representing a better QoL. VHI-10-scores are obtained with the Voice Handicap Index 10, range 0 to 40, with a higher score representing more voice handicap. *AVQI:* Acoustic Voice Quality Index scores range 0–10, a lower score indicating better acoustic voice quality; Perc. Voice SLP: Perceptual rated voice quality of the participant rated by the speech language pathologist (visual analogue scale 0–10); Perc. Voice Pt: Perceptual rated voice quality by the participant (visual analogue scale 0–10), for the perceptual scales a higher score indicates better perceptual quality

**Fig. 3:**
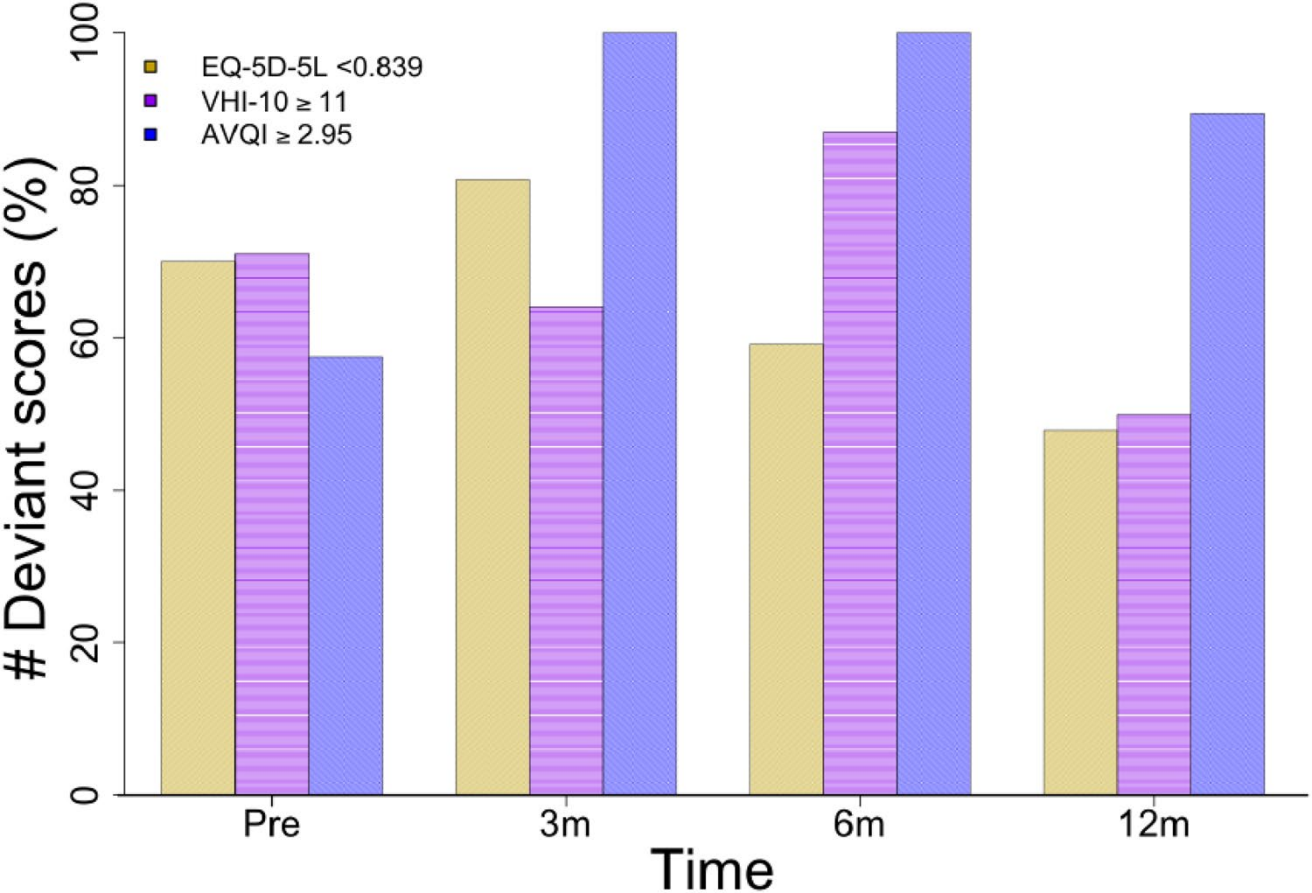
Graph visualizing participants (%) with unsatisfactory (abnormal) scores over time for *EQ*-*5D*-*5L*, *VHI*-*10*, and *AVQI*

Worst mean *EQ*-*5D*-*5L* values are reported at T0, mean 0.712 (SD 0.203) (Table 4). Over time a gradual improvement of mean *EQ*-*5D*-*5L* values are seen. At T3 the mean *EQ*-*5D*-*5L* value is 0.830 (SD 0.164), which is equivalent to the reference value of 0.839 (SD 0.179) determined for the Dutch general population aged 60–70 years [23]. Before surgery 70% of the participants report a score lower than this reference value, Fig. 3 shows that there is an improvement in participant reported scoring with only 48% of participant scores being outside normal limits.

Mean values for *VHI*-*10* were at all assessment time points above 11, which is indicated as disordered [26]. Before surgery participants report a mean *VHI*-*10* score of 16.7 (SD 10.6), this worsens at T1 with a mean score of 20.3 (SD 10.0), and over time gradually improves back to baseline level at T3 with a score of 15.8 (SD 12.0). Figure 3 visualizes the percentage of participants reporting a score above 11, which is before surgery 71%, At T2 87%, declining to 50% at T3. When investigating individual course, a response shift is seen after surgery; Some participants expressed they were happy to be alive and satisfied with the fact that they can express themselves verbally, they indicate the quality of the sound of the voice as less important, whilst during pre-surgery assessment their *VHI*-*10* was clearly impaired.

The mean AVQI score rising from 3.57 (SD 1.69) at T0 to 8.07 (SD 2.77) at T2 indicates a clear deterioration of acoustic voice quality after surgery (Fig. 2). At all assessment time points, participants voices are dysphonic, with a mean AVQI score above 2.95. AVQI scores remain impaired and never reach baseline level again. Figure 3 shows that 58% of the participants have an AVQI score indicating impairment at T0, this increases to 100% at T1 and T2. At T3, numbers are still increased to 90% of the participants.

A similar course of perceptual outcome evaluations by the clinician and the participant are found, T0 scores are: *Perc. Voice SLP* score 4.65 (SD 2.58), and *Perc. Voice Pt* score 4.65 (SD 2.58), deterioration is seen at T1, gradually improving over time. At the time-points T1–T3 mean *Perc. Voice SLP* scores are consistently about 0.5 points higher compared to the perceptual evaluation of the participant.

### Correlations between outcome measurements

When outcomes for the multidimensional assessment methods assessed over time are pooled, strong correlations are found between the dimensions of voice-related outcomes. Correlations between the multidimensional voice-related outcomes are statistically significant (see Appendix I). No statistically significant correlation is found between *AVQI* and *EQ*-*5D*-*5L* (*p* = 0.228).

Correlations between the outcome measurement instruments for the post-surgery time-points (T1–T3) show statistically significant correlations between voice-related outcomes (see Appendix II). Statistical significance is lost for voice-related outcomes with QoL, seen in the correlation between *VHI*-*10* and *EQ*-*5D*-*5L* (*p* = 0.021), and *AVQI* and *EQ*-*5D*-*5L* (*p* = 0.467). *AVQI* and *VHI*-*10* (*p* = 0.017) still correlate strongly but this is not statistically significant with the statistical level set at *p* ≤ 0.01. With the investigation of the correlation between *VHI*-*10* and *AVQI* for the post-surgery time points (T1–T3) an *AVQI* cut-off score of 6 would be indicative for a *VHI*-*10* score > 11, indicating an unsatisfactory voice after total laryngectomy.

### Predictors of voice outcome

We created linear mixed-effects models to explore the effect of *Treatment*, *Reconstruction*, and *Time* studied for the main voice outcomes VHI-10, AVQI, *Perc. Voice SLP*, and *Perc. Voice Pt*. *Time* is indicated as a significant factor in predicting *AVQI* score (*p* ≤ 0.001), *Perc. Voice SLP* (*p* = 0.015), *Perc. Voice Pt.* (*p* = 0.002) but not for *VHI*-*10* (*p* = 0.368). Modelling predicted outcomes for the groups for (a) *primary surgical treatment vs. salvage surgical treatment* and (b) *primary closure vs. major reconstruction* did not reach statistical significance.

## Discussion

In our study, quality of life, measured with *EQ*-*5D*-*5L*, is lowest before surgery. It is known that levels of anxiety and self-care are severely impacted before as well as up to 14 days post-surgery [7, 30, 31]. We observe improvement at the 3 months post-surgery assessment, it is likely that patients have begun to adjust to their condition compared to 14 days post-surgery. In the long term, at 12 months post-surgery, mean score for the total group is comparable to reference values for the age group above 60 years old [23]. This positive result might be influenced by drop-out of patients who were excluded due to mortality, nevertheless, it indicates that the remaining patients are fairly well adjusted to their condition. This general course of worsening after surgery and gradual improvement over time corresponds to findings of earlier studies assessing the course of QoL [7, 8].

Mean values for *VHI*-*10*, were at all assessment time points above 11, which is indicated as having a voice handicap [25, 26]. This is in line with earlier studies showing patient-reported voice problems as a result of tumour presence, tracheostomy and earlier organ sparing oncologic treatment, as well as after total laryngectomy [2, 32, 33]. It is acknowledged that the VHI and VHI-10 are not specifically validated for use after total laryngectomy. A study of Moerman et al. has introduced a corrected VHI score (30 item version) specifically to use after TL, which copes with unanswered items [34]. This is useful since not all questions apply after TL. Future studies could develop this corrected score for the VHI-10, validate the instrument for use after TL, and determine a cut-off score.

The acoustic voice outcomes, measured with *AVQI*, are impaired at all time-points. However, we found a significant deterioration after total laryngectomy. Both t test (pre- to 6 months post-surgery) and Linear Mixed Effect modelling showed statistical significance (both *p* < 0.001 resp.) Earlier research showed a strong correlation between AVQI and perceptual rated voice quality [35]. This study again shows a strong correlation between *AVQI* and *perceptual rated voice quality*, as well as between *AVQI* and *VHI*-*10*, indicating that these tools measure the same construct. With the confirmation of the *AVQI* correlating to *perceptual outcomes*, as well as detecting differences over time, there is justification for *AVQI* use in TE-speech samples [4, 36]. In this study, an *AVQI* score of ≥ 6 correlates with a *VHI*-*10* score > 11. This cut-off should be validated in a larger study.

We find a statistically significant effect of time in perceptual outcome evaluations of voice quality by the clinician as well as the participant (LME model). There is a clear deterioration in perceptual rated voice quality and intelligibility after surgery followed by a gradual improvement over 12 months.

No effect is found for the investigated oncologic treatment variables *a) primary surgical treatment vs. salvage surgical treatment* and b) *primary closure vs. major reconstruction*. It is known that oncological history of CRT negatively influences complication rates including fistula, and stricture [37, 38], but we found no influence on QoL or voice outcomes. Earlier literature showed inferior voice quality in patients with total laryngectomy who received a major reconstruction of the neopharynx [39]. Previously, Jacobi et al. also reported optimal voice characteristics in tubed flap reconstructions [40]. This shows that the voice after flap reconstruction can be comparable as after primary closure. However, we could not confirm that the low number of patients did not allow us to look at specific reconstruction techniques.

### Strengths and limitations

To our knowledge, this is the first study prospectively assessing a combination of acoustic, patient rated, and clinician-rated voice outcomes from pre-up to 12 months post-surgery. The prospective character of the study aims to overcome a selection bias of including only excellent speakers. The unique approach with assessing acoustic, self-reported and perceptual outcomes over time provides information about the course of voice outcome and QoL. With the combination of instruments which are used, effectiveness and responsiveness of the instruments for changes over time are evaluated. By conducting this study in five hospitals in two countries, a variety of patients, languages, and treatment strategies are involved. We evaluated the effects of medical detail on voice outcome, and although the number of participants of our study led to no significant results in medical history factors, this framework is useful for ongoing work.

This study has some limitations. Due to the small sample size, multiple assessments, and the variety of outcome measures we were forced to perform the LME modelling on summarized dichotomized variables. With restructuring variables into dichotomous variables information about details in the surgery are lost, e.g. *Major Reconstruction* is used as a summarized variable which originally included details on type and extent of (flap) reconstruction. Although all evaluation tools are widely used, they are not validated for use after TL. By conducting this study as a prospective cohort study, we aimed to overcome selection bias; nevertheless, a number of participants were not included, assessments were missed due to logistic reasons and medical complications, and participant mortality were excluded from the study. Therefore, outcomes are collected from patients who are alive and willing to fulfil study-related procedures, which may lead to overestimation of the outcomes. We anticipated on evaluating different voice methods, e.g. esophageal speech and electrolarynx speech. In this cohort, however, no esophageal speakers were present and only two participants used electrolarynx speech. Therefore, no sub-group analysis between voice methods could be performed.

### Recommendations for clinical practice and future research

Thirty per cent (*n *= 13) of participants did not complete the study due to mortality. Sadly, nine participants did not reach acceptable (TE-)speech and had to depend on augmentative alternative communication methods such as typing, writing and mouthing in the palliative phase of their life. For clinical practice, it is recommended to inform patients about the possibility to end-up without sufficient TE-speech, especially when the prognosis is poor.

The instruments in this study have shown to be useful to detect a difference over time from pre- to 1-year post-surgery. Former studies that have evaluated voice outcomes after total laryngectomy utilise a wide variety of measurement tools and time points after surgery [2, 4]. *AVQI*, *VHI*-*10*, *EQ*-*5D*-*5L*, and VAS scales for *perceptual ratings*, used in our study, proved to be sensitive to detect differences over time from pre- to post-surgery. Sensitivity is lacking when differences between treatment groups and over time post-surgery have to be detected. Continued efforts are needed to establish the optimal tools, and validate these instruments for research and clinical practice in this population.

Improvement for patient-reported voice functioning and QoL at 12 months post-surgery was found, whilst AVQI score remains altered (Fig. 3). This could be interpreted as a response shift with a change of internal standards, values, and meaning of QoL [41]. The response shift could be explained by the ability of human beings to adapt to life events. Investigating this response shift specific to the head and neck cancer group is an important issue for further research. To develop a full picture of what speech-related QoL means for individuals before and after a total laryngectomy we suggest to perform studies with a combination of acoustic, patient-rated, and clinician-rated methods, to explore how speech-related QoL is related to these measures.

## Conclusion

Outcomes show that voice-related outcomes are already impaired before surgery, all worsen after surgery with a gradual improvement from 6 up to 12 months post-surgery. A response shift is seen in *VHI*-*10*, were acoustic measured voice quality worsen, reported voice handicap indicates acceptance of the condition and sufficient coping in the long term.

The study leads to recommendations for clinical practice; before total laryngectomy, patients should be counselled on the expected course of voice problems after surgery, with a focus on the long-term acceptable outcomes which are reached in TE-speakers. The discrepancy between reported voice handicap and objective acoustic rated voice quality, clearly demonstrates that a patient’s adjustment to post-laryngectomy dysphonia does not solely rely on their acoustically measured voice quality. As such, clinicians should utilise a range of measures—both acoustic (instrumental) and patient or clinician reported, to comprehensively analyse a patient’s vocal ability. Lastly, patients should be prepared for the possibility that they might not accomplish acceptable TE-speech during their post-treatment phase, especially when medical complications occur, or oncologic treatment fails. This may be more common in the salvage procedures.

The findings of this study have implications for future research. A specific *AVQI* cut-off value for TE-speech should be determined, as well as assessing the discriminative power of this instrument in this type of speech. Validation of the *VHI*-*10* specifically for use after total laryngectomy is needed. We demonstrate a change in response of patient-reported outcomes after total laryngectomy in relation to acoustic outcomes. Patient-reported outcome measures reflect the way patients accept their condition and cope with their permanent altered speech. This is likely to vary depending on their access to support (medical, nursing and allied health, funding and equipment, support of family and friends). Future research in vocal functioning after total laryngectomy should expand beyond vocal impairment, evaluating psychosocial consequences and participation restrictions. Simultaneously, investigating the effect of medical history, including oncologic treatment factors on voice outcome, can ultimately lead to personalized pre-surgery counselling.

## Figures and Tables

**Fig. 4:**
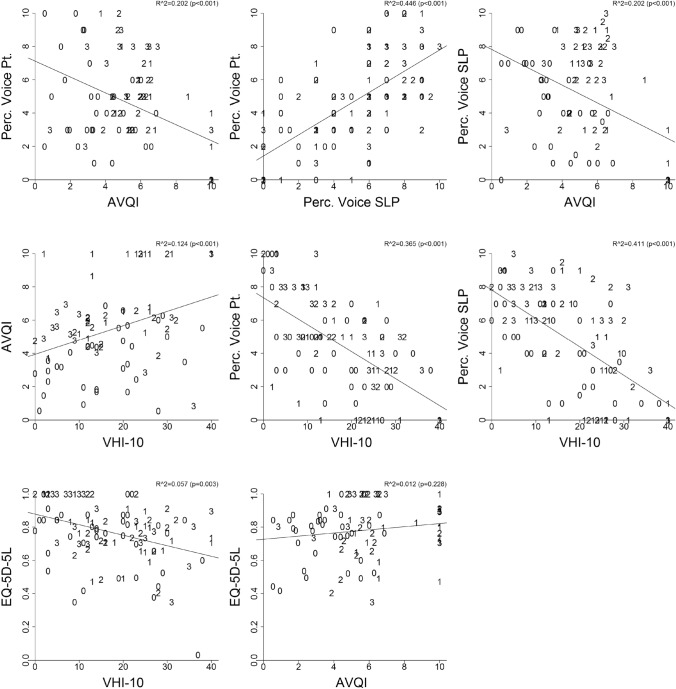
Correlation between outcome parameters for all time points. Numbers indicate T (T0–T3). *Y* ~ *X* + (1|Subject) *R*^2^: pseudo *R*^2^ (explained variance fixed factors); *p*: > Chi square (ANOVA vs *Y* ~ 1 + (1|Subject)). Abbreviations: 0 = pre-laryngectomy; 1 = 3 months post-laryngectomy; 2 = 6 months post-laryngectomy; 3 = 12 months post-laryngectomy

**Fig. 5:**
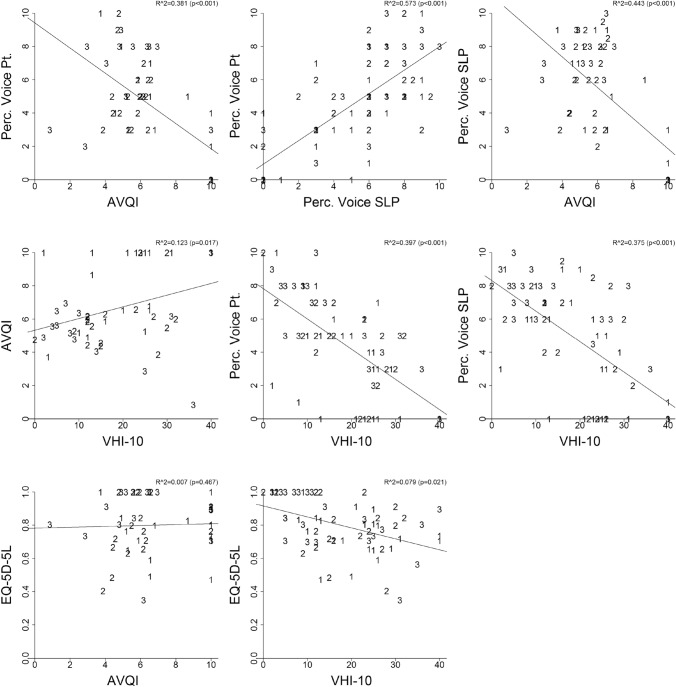
Correlation between outcome scores for the post total laryngectomy time-points. Numbers indicate T (T1–T3). *Y* ~ *X* + (1|Subject) *R*^2^: pseudo *R*^2^ (explained variance fixed factors); *p*: > Chi square (ANOVA vs *Y* ~ 1 + (1|Subject)). Abbreviations: 0 = pre-laryngectomy; 1 = 3 months post-laryngectomy; 2 = 6 months post-laryngectomy; 3 = 12 months post-laryngectomy

**Fig. 6:**
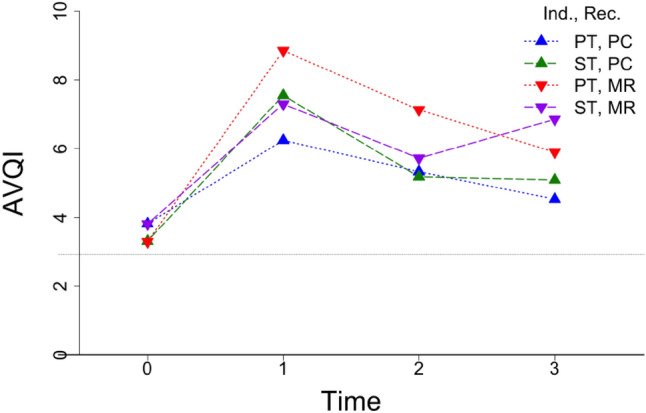
AVQI as a function of time point and treatment. AVQI ~ Time * Ind. * Rec. Pseudo *R*^2^: 0.440 (*R*^2^m fixed factors), 0.519 (*R*^2^m complete model). ANOVA: ~ 1 vs Time: *p* < 6.28e−12. Ind.: indication, Rec.: reconstruction, PT: primary surgical treatment, ST: salvage surgical treatment, PC: primary closure, MR: major reconstruction. Time points: 0 = pre, 1 = 3 m, 2 = 6 m, 3 = 12 m

**Fig. 7:**
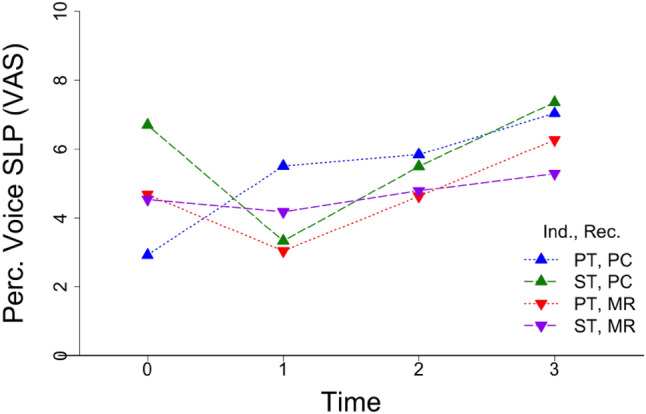
Perceptual Voice evaluation as a function of time point, reconstruction, and treatment indication. SLP Voice ~ Time * Ind. * Rec. Pseudo *R*^2^: 0.147 (*R*^2^m fixed factors), 0.434 (*R*^2^m complete model). ANOVA: ~ 1 vs Time: *p* < 0.015. Ind.: indication, Rec.: reconstruction, PT: primary surgical treatment, ST: salvage surgical Treatment, PC: primary closure, MR: major reconstruction. Time points: 0 = pre, 1 = 3 m, 2 = 6 m, 3 = 12 m

**Fig. 8:**
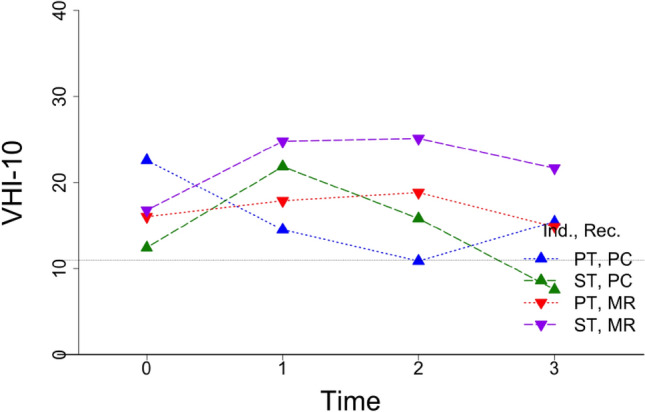
VRQOL score as a function of time point and treatment. VRQOL score ~ Time * Ind. * Rec. Pseudo *R*^2^: 0.168 (*R*^2^m fixed factors), 0.281 (*R*^2^m complete model). ANOVA: ~ 1 vs Time: *p* < 0.368. Ind.: indication, Rec.: reconstruction, PT: primary surgical treatment, ST: salvage surgical treatment, PC: primary closure, MR: major reconstruction. Time points: 0 = pre, 1 = 3 m, 2 = 6 m, 3 = 12 m

## Appendices

### Appendix I

Correlations between the outcome measurement instruments for all time points (T0–T3) show statistically significant correlations between AVQI and VHI-10 (*p* < 0.001), Perc. Voice SLP and VHI-10 (*p* < 0.001), Perc. Voice pt and VHI-10 (*p* < 0.001), Perc. Voice pt and AVQI (*p* < 0.001), Perc. Voice pt and Perc. Voice SLP (*p* < 0.001), Perc. Voice SLP and AVQI (*p* < 0.001) and between VHI-10 and EQ-5D-5L (*p* = 0.003). No statistically significant correlation is found between AVQI and EQ-5D-5L (*p* = 0.228) (Fig. 4).

### Appendix II

Correlations between the outcome measurement instruments for post-operative time points (T1–T3) show statistically significant correlations between Perc. Voice SLP and VHI-10 (*p* < 0.001), Perc. Voice pt and VHI-10 (*p* < 0.001), Perc. Voice pt and AVQI (*p* < 0.001), Perc. Voice pt and Perc. Voice SLP (*p* < 0.001), and Perc. Voice SLP and AVQI (*p* < 0.001). No statistically significant correlation is found between AVQI and EQ-5D-5L (*p* = 0.228), AVQI and VHI-10 (*p* < 0.017), and between VHI-10 and EQ-5D-5L (*p* = 0.021) (Fig. 5).

### Appendix III

See Fig. 6.

### Appendix IV

See Fig. 7.

### Appendix V

See Fig. 8.
